# Supplementary material for: Neutrophil-to-lymphocyte ratio: a potential supportive marker for elderly community-acquired bloodstream infections—a retrospective study
Source: PeerJ. 2025 Dec 8;13:e20449. doi: 10.7717/peerj.20449 (PMC12697295; doi:10.7717/peerj.20449)
Supplement: Supplemental Information 2 [file peerj-13-20449-s002.docx]

translations of non-English text

location: Row 52, Column 78

Chinese:“单瓶血培+”

translation:Blood Culture Positive?

location:Title at the lower left corner of the table

Chinese:“spss输入表（固定） (2)”

translation:SPSS input table
